# Pilot implementation of the national longitudinal communication curriculum: experiences from four German faculties

**DOI:** 10.3205/zma001448

**Published:** 2021-03-15

**Authors:** Barbara Hinding, Christian A. Brünahl, Holger Buggenhagen, Nadine Gronewold, Anke Hollinderbäumer, Kirsten Reschke, Jobst-Hendrik Schultz, Jana Jünger

**Affiliations:** 1 Institut für Medizinische und Pharmazeutische Prüfungsfragen (IMPP), Mainz, Germany; 2 Universitätsklinikum Hamburg-Eppendorf, Institut und Poliklinik für Psychosomatische Medizin und Psychotherapie, Hamburg, Germany; 3 Universitätsklinikum Hamburg-Eppendorf, Klinik und Poliklinik für Urologie, Hamburg, Germany; 4 Universitätsmedizin der Johannes Gutenberg-Universität Mainz, Rudolf Frey Lernklinik – Zentrale Lehrplattform, Mainz, Germany; 5 Universitätsklinik Heidelberg, Klinik für Allgemeine Innere Medizin und Psychosomatik, Heidelberg, Germany; 6 Otto-von-Guericke-Universität Magdeburg, Klinik für Nieren- und Hochdruckkrankheiten, Diabetologie und Endokrinologie, Magdeburg, Germany

**Keywords:** education, curriculum, implementation science, communication, patient-centered care

## Abstract

**Background: **The German national longitudinal communication curriculum provides medical faculties with orientation for the content of their communication teaching. But its implementation also requires changes in the organization of teaching. However, due to a lack of reports and studies on experiences with the development and implementation of communication curricula, recommendations on the procedure and the use of suitable instruments cannot be provided. Consequently, as part of this exploratory study the implementation process of the communication curricula was observed at four faculties.

**Methods:** A comparative case study was conducted against the background of a change management concept. The four participating faculties were selected in such a way that they differed significantly in their initial conditions, particularly the development stage of the communication curriculum. Group interviews were conducted with the project teams at each faculty concerning the conditions and experiences with the implementation process. The evaluation took the form of a qualitative content analysis with a focus on identifying supporting and inhibiting factors and useful activities.

**Results: **Different faculty approaches, support structures, core skills, the scope of study and examination regulations, teaching organization and available resources can have a major impact on the implementation processes. It became clear that, depending on the status of the implementation process, other barriers and supporting conditions gain in importance. Strategically, it proved to be a particular success factor to implement the communication curriculum together with other innovations in the course of the conversion to a model study program. This enabled a particularly quick and efficient implementation. The implementation into an existing curriculum proved to be much more protracted. In addition, a change management concept was used to illustrate which measures were found to be effective for which task areas. This includes, for example, curricular mapping, the development of skills in communication teaching or the integration of communication into exams.

**Conclusions: **Thus a concept with strategies and measures for the implementation of the National Longitudinal Communication Curriculum is available. It already contains numerous suggestions for planning one's own approach in line with the conditions and resources of other locations. However, it needs to be supplemented and further validated.

## Zusammenfassung

**Hintergrund: **Das Nationale longitudinale Mustercurriculum Kommunikation gibt medizinischen Fakultäten Orientierung für die inhaltliche Gestaltung ihrer Kommunikationslehre. Zu seiner Umsetzung sind jedoch auch Veränderungen in der Organisation der Lehre erforderlich. Bisher fehlen allerdings Berichte und Untersuchungen zu den Erfahrungen mit der Entwicklung und Implementierung von Kommunikationscurricula, aus denen Empfehlungen zum Vorgehen abgeleitet werden können. Daher wurden im Rahmen dieser explorierenden Untersuchung vier Fakultäten bei der Implementierung ihrer Kommunikationscurricula begleitet.

**Methoden: **Vor dem Hintergrund eines Change-Management-Konzepts wurde eine vergleichende Fallstudie durchgeführt. Die beteiligten vier Fakultäten wurden so ausgewählt, dass sie sich in ihren Ausgangsbedingungen, insbesondere im Entwicklungsstand des Kommunikationscurriculums, deutlich unterschieden. An jeder Fakultät wurden Gruppeninterviews mit den Projektteams zu den Voraussetzungen und den Erfahrungen mit dem Implementierungsprozess durchgeführt. Die Auswertung erfolgte in Form einer qualitativen Inhaltsanalyse zur Identifikation unterstützender und hemmender Einflussfaktoren und zielführender Aktivitäten.

**Ergebnisse: **Einstellungen an der Fakultät, Unterstützung, Kompetenzen, der Spielraum der Studien- und Prüfungsordnungen, die Lehrorganisation und die verfügbaren Ressourcen können großen Einfluss auf Implementierungsprozesse nehmen. Deutlich wurde, dass je nach Stand im Implementierungsprozess andere Barrieren und unterstützende Bedingungen im Vordergrund stehen. Strategisch erwies es sich als ein besonderer Erfolgsfaktor, das Kommunikationscurriculum im Zuge der Umstellung auf einen Modellstudiengang gemeinsam mit anderen Innovationen umzusetzen. Dadurch war eine besonders rasche und effiziente Umsetzung möglich. Die Implementierung in ein bestehendes Curriculum erwies sich als deutlich langwieriger. Zusätzlich wurde anhand eines Change-Management-Konzepts abgebildet, für welche Aufgabenfelder welche Maßnahmen als zielführend befunden wurden. Dazu gehört etwa das Curricular Mapping, die Entwicklung von Kompetenzen zur Kommunikationslehre oder die Integration von Kommunikation in die Prüfungen.

**Schlussfolgerungen: **Somit liegt ein Konzept mit Strategien und Maßnahmen zur Implementierung des Nationalen longitudinalen Kommunikationscurriculums vor. Es enthält bereits jetzt zahlreiche Anregungen zur Planung des eigenen Vorgehens passend zu den Gegebenheiten und Ressourcen anderer Standorte. Allerdings bedarf es der Ergänzung und weiteren Validierung.

## 1. Introduction

Communication and conversation are a central component of medical activity. The importance of their quality for the well-being, satisfaction, willingness to cooperate and the health of patients has been proven many times over [1], [2], [3], [4], [5], [6]. This makes it all the more important to train communication skills during medical school [7], [8]. In recent years, various faculties have developed curricula for integrating basic communication skills with patients into medical training [9], [10], [11], [12], [13], [14]. As part of the project “National Longitudinal Model Curriculum for Communication in Medicine”, funded by the German Federal Ministry of Health, an interdisciplinary and interprofessional working group with representatives from all 36 medical faculties in Germany developed a model curriculum [15], [16]. It is based on the National Competence-Based Catalogue of Learning Objectives in Medicine (NKLM), which defines learning objectives for medical conversation in a subchapter [http://www.nklm.de], [17]. The “National Longitudinal Model Curriculum for Communication in Medicine”, assigns a time frame to each learning goal, adding to 300 teaching units (TU) for medical conversation [18]. This sample curriculum was explicitly named as measure <8> in the Master Plan for Medical School 2020.

The present study examines which factors support the implementation of the curriculum. In the faculties, this requires strategies that take existing conditions and resources into account. We therefore want to achieve a better understanding of the process of implementing communication curricula. This is an exploratory study with qualitative methods based on the design-based research approach [19], [20]. 

Four medical faculties were supported academically in the implementation of the communication curriculum. In 2014, all four took part in a survey of communication studies at 31 German medical faculties (“Longkomm Project”) [18], [21]. This made it possible to select the four faculties so that they were at different stages of implementing their communication curriculum. In 2018, another survey of the communication curriculum was carried out and the previous approach to implementing the then existing curriculum was retrospectively reflected with regard to success factors and inhibiting conditions. The results presented here then served as a starting point for an intensified implementation phase in which the existing communication curriculum was further developed in accordance with the “medical conversation” component of the model curriculum [18]. 

## 2. Theoretical basics

In order to describe the changes between 2014 and 2018, reference was made to the maturity level concept of communication curricula. It postulates a prototypical course of the implementation process and provides evaluation criteria for assessing the development status of communication curricula [22]. Thus, the concept also formulates tasks which arise in the context of a curriculum implementation. The execution of these tasks was viewed as management of a change process with several steps. “Change management” has its origins in the phases of change described by Lewin [23]. On this basis, Kotter [24], [25] identified factors critical to success by examining failed change processes, from which he derived eight steps for designing change processes in the form of a recommendation (see table 1 (Tab. 1)). 

All steps are associated with different tasks, whose success can depend on a range of factors. For instance, the creation of a guiding coalition can mean to set up an influential team at the faculty.

A consistent research result in change management is the importance of initial “institutional readiness”. This refers to the extent to which the organization and its members are collectively willing, motivated, and able to implement the change measure [26], [27], [28]. In this case this means the readiness of the faculties and their members to implement the communication curriculum and the conditions that the faculty provides for this. This addresses both individual and structural factors that promote or impede the implementation process [29].

## 3. Methods

### 3.1. Description of curriculum changes

**Figure 1:**
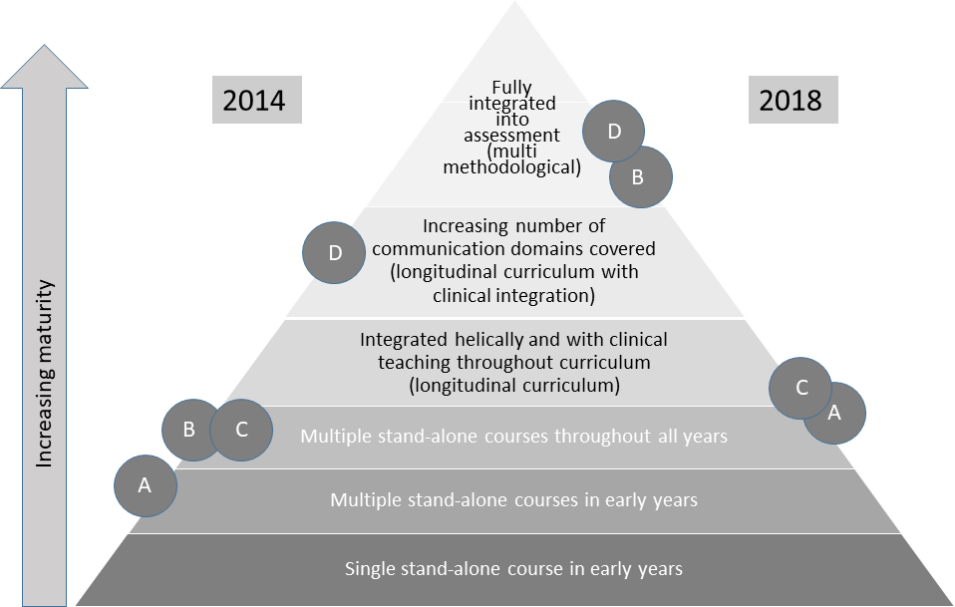
Change in the position of the examined faculties on the pyramid of increasing maturity of communication curricula. Own illustration according to Silverman [22].

Silverman [22] describes the degree of maturity of a communication curriculum in six qualitatively distinguishable levels. These are arranged in a pyramid (see figure 1 (Fig. 1)), with the level of maturity increasing from bottom to top. At the beginning, at the lowest level of maturity, communication is only taught in the first years of study in a single course. At the next level, the number of courses increases, but they still only take place in the first years of study. Extending the teaching to the end of the academic program is at level three. Level four represents a real qualitative leap, where there is a longitudinal curriculum that is helically integrated (building on each other in learning spirals) and is integrated into the clinical curriculum. At level five, this process continues until all the intended contents are covered. At the end, at level six, communication is fully represented in the examinations.

The first classification on the pyramid was based on the results of the survey of 31 medical faculties conducted in 2014. The faculty teachers who teach communication-related content provided information about their courses in partially standardized interviews. 

Among other things, the first classification of the four faculties was based on the results of the survey of the 31 medical faculties conducted in 2014. The faculty teachers who teach communication-related content provided information about their courses in partially standardized interviews. 

The following was considered for the assignment to a level:

- the number of courses with communication-related content
- the semester in which the course is to be taken
- the learning objectives covered
- the assignment of the course to the subjects.

In addition to these quantitatively determined variables, consultation was held with the project managers at the locations to determine,

- whether there is a coordinated longitudinal curriculum with TU based on each other,
- the extent to which communication is integrated into subject instruction,
- whether there is a coherent approach to communication testing.

All four faculties had at least several individual courses in the first academic years and over the entire period of study (without PY) (see table 2 (Tab. 2)), i.e. they were at least at level 2 or 3. Assignment to level 4 requires a longitudinal concept and the integration of communication-related content into clinical teaching. Such a concept existed only at site D. With regard to level 5, the learning objectives defined in the NKLM/sample curriculum were considered. Only Faculty D covered these to a large extent. Examinations with communication content existed at all participating faculties, but there was no coordinated overall concept integrated into the subjects.

Based on this data, a preliminary assignment of the curricula to the maturity levels was made and coordinated with the project managers at the locations. The result is shown on the left side of figure 1 (Fig. 1). 

The survey was repeated at the beginning of 2018 at the four participating faculties. It showed that in the meantime the level of maturity increased by at least one level in all four faculties, and by three in one of them. This can be seen on the right side of figure 1 (Fig. 1). 

#### 3.2. Survey and analysis methods

With this result in mind, those responsible for the project at the faculties were asked about their approach and experiences up to 2018. It was determined specifically for each location how the development of the communication curriculum had proceeded so far and which factors had favored the implementation and which had possibly delayed it.

For this purpose, a group discussion of one hour duration was conducted at each participating faculty [30], [31]. In addition to two interviewers, the project managers and team members took part. They were two people in Faculty A, two people in Faculty C, five people in Faculty B, and two people in Faculty D. 

The interview guidelines contained open questions on the strengths and weaknesses of the faculty in terms of the implementation of the communication curriculum and on institutional readiness (Attitudes towards teaching communication, existing skills, support, financial resources). They were also asked about the implementation measures that have been taken. For each of the eight change management steps (see table 1 (Tab. 1)), participants were asked to indicate how they had been implemented so far and what had helped them. 

The discussions were recorded and then subjected by the two interviewers to a structuring content analysis [32], separated according to promoting and inhibiting conditions as well as goal-oriented activities. The statements were paraphrased and grouped according to similarity. This resulted in six categories of promoting and inhibiting factors. Likewise, the mentioned activities were assigned to each of the eight steps, redundancies eliminated, and similar contents grouped into generic terms. After the categories had been defined, the text passages were reassigned to the categories by one of the interviewers and another person who had not previously been involved in the evaluation process, with the aim of reliability testing. The evaluator agreement is 83.9% for the institutional framework and 85.3% for the goal-oriented activities.

## 4. Results

### 4.1. Promoting and inhibiting conditions in the implementation process

The comparison of the faculties in terms of promoting and inhibiting conditions revealed considerable similarities in content at similar levels of maturity, so that faculties A and C as well as B and D were combined (see table 3 (Tab. 3)).

The faculty members’ attitudes towards communication teaching were described as an influential factor with immense significance. If the majority considered communication to be of low importance, this was considered a major obstacle at low levels of maturity. But conflicts of interest and attitudes against more communication instruction were also reported at high levels of maturity.

If the degree of maturity is low, the support of the teams by the faculty management, the teaching coordinators of the individual subjects, and committed students is particularly critical for success. With a high degree of maturity this kind of support is given, but must be secured again and again, e.g. because central persons migrate. 

The skills available at the faculty for communication teaching increase with the degree of maturity. At the beginning, there were only a few lecturers qualified to do so. They, however, contributed decisively to implementation. At a higher level of maturity, targeted competence development was reported through ongoing training, support for MME students, and the expansion of teaching-learning research. 

Another success factor was seen in the integration of communication teaching into quality management and teaching evaluation. The continuous student feedback increases competence in the field of communication throughout the faculty.

Regardless of the degree of maturity, the study and examination regulations are often seen as an obstacle because they contain little room for innovation, especially for new types of examinations. The decentralized teaching organization, in which the individual departments are responsible for the implementation of communication teaching independently of each other, is also perceived as an impeding factor. 

#### 4.2. Goal-oriented activities within the framework of change management 

The first analysis of the measures and activities for the implementation of the curriculum showed that if the maturity level of the communication curriculum was low, steps 1-6 were most often worked on, while steps 7 and 8 were worked on if the level of maturity was high. Table 4 (Tab. 4) shows those activities that have proven successful from the perspective of the interviewees.

In the first step, “Establishing a sense of urgency”, the opportunity of referring to educational policy developments to promote the communication skills of prospective doctors and the discrepancy with the status quo at the faculty was considered particularly helpful. The systematic inventory of the teaching content on communication, the curricular mapping, which shows gaps and redundancies, proved to be extremely helpful in this respect.

For the second step, “Creating the guiding coalition”, it was emphasized how difficult it was to recruit influential members of the faculty management for the implementation team at the beginning. The significance of this was particularly evident where support was promised but not provided through active involvement. Equally important was establishing a good internal network, as it helps new projects experience greater acceptance and makes upcoming planning and coordination processes easier to manage.

Step 3 refers to the vision and strategy. The most important result is that the overall strategy is of crucial importance. It makes a big difference whether the communication curriculum is inserted into an existing curriculum or is integrated as part of a more extensive redesign of the program. The enormous leap in the maturity of the communication curriculum of Faculty B can be attributed primarily to the fundamentally different strategic approach. There, a model academic program was developed in which the communication curriculum was planned from the beginning and implemented together with other changes. 

The implementation into an existing curriculum during ongoing operations is more complex and time-consuming. In this case, a step-by-step approach is required, in which the initial curricular mapping was considered particularly helpful. In addition to gaps and redundancies in the content, it provided information about horizontal and vertical integration and coherence: Is the communication topic being dealt with simultaneously in courses of different subjects and is this coordinated in a meaningful way? Do the contents and methods build on each other over the course of the academic program? Is what is taught also tested? 

A particular difficulty was that the departments do not know what communication content is taught by whom and how. This results in redundancies and inconsistencies, for example different terminology and the lack of a longitudinal system, so that extensive coordination processes are necessary. A centralized teaching organization, e.g. with a platform on which information about courses is brought together, was considered a very valuable tool for coordination. 

A particular difficulty was that the departments do not know from each other which communication content is taught by whom and how. The consequences are redundancies and inconsistencies, for example different terminology and the lack of a longitudinal system, so that extensive coordination processes are necessary. A central teaching organization, e.g. with a learning platform, through which information about courses etc. is brought together, was seen as a very valuable tool for coordination.

Communicating the vision of change (step 4) and soliciting acceptance, the arguments listed in the table have proven helpful. In later phases of the implementation process, it is advisable to seek a broader public to raise awareness of the topic at the university and beyond.

To create a broad implementation basis (step 5) the qualification of the teaching staff is the central task. In view of limited resources, the possibility of standardized, certified training of student tutors was considered highly efficient (see [33], [34]). 

The participation of an increasing number of people in the process requires the establishment of structures, e.g. regular meetings or access to best practice examples, such as the toolbox, a moderated platform on which tutors can exchange teaching and testing examples [35]. 

The lack of simulation persons was highlighted as a particularly significant obstacle to the realization of competence-oriented teaching. Since they allow for practice in practical learning scenarios as well as the implementation of realistic exams, the creation of a pool of actors was considered absolutely necessary in order to implement communication theory according to the current state of knowledge. 

The generation of short-term wins (step 6) is provided, for example, by pilot events that are announced, evaluated, and published throughout the faculty. The presentation of progress in communication theory at the faculty and the public recognition of good examples were also mentioned here. 

Steps 7 and 8, consolidation and anchoring in culture, were combined because no group interview explicitly described specific measures for anchoring communication or medical conversation in culture. The inclusion of communication examinations in the faculty’s examination plan was mentioned as an essential step towards consolidation. 

The faculties with a higher degree of maturity emphasized that a curriculum is never really “finished”. Continuous adjustments to new developments are necessary. Triggers can be external requirements, but also the results of internal quality management and evaluation, which give rise to permanent improvements. As a result, the scope and complexity of the curriculum increases, making it necessary to fill long-term positions for coordination.

## 5. Discussion and conclusions

Progress in the maturity of the communication curriculum was noted in all four participating faculties. Faculty B stands out in particular, which, starting from multiple individual courses, has achieved an integrated curriculum with exams in a period of 3-4 years. This was made possible by the development of a model study program in which communication was a central element. Through this strategy, implementation took place top-down and bundled with other measures, quite unlike in the three other faculties, where the initiative of committed individuals or small groups came predominantly from intermediate structures. 

At the time of the second survey, Faculties A and C were faced with the task of integrating multiple individual courses into a longitudinal curriculum, while Faculties B and D were in the process of consolidating, completing, and updating what they had achieved. Thus, according to the terminology of change management, different steps had to be taken. Relatively at the beginning, the focus lies on team building, planning, persuasion, and broadening implementation possibilities. The later phases focus more on safeguarding and further development the existing. 

The longitudinal and interdisciplinary integration of the curriculum was described as particularly challenging across faculties (Level 4 in figure 1 (Fig. 1)). The task consists of locating the individual teaching units on communication in the subject courses and at the same time distributing them over the entire academic program in such a way that they meaningfully build on each other. This step involves a great deal of organization and coordination. The following measures proved to be particularly successful in this process:

- Curricular Mapping, with its various functions for attitude change and strategy development [36], [37]
- Competence development: training of lecturers and of student tutors and their integration into teaching [33], [34]
- Faculty-internal networking to facilitate cooperation and coordination.

For advanced levels of maturity, the focus is on consolidation. The following points were considered to be particularly helpful in this respect:

- Integration into examination plan/examination regulations, because: assessment drives learning [38], [39]
- Integration of communication teaching into teaching evaluation and quality management as the basis for continuous adaptation and improvement
- Teaching-learning research to develop further specific expertise.

All in all, the results provide a multifaceted picture of the conditions, activities, and measures that can be of importance in the course of an implementation process. As we have seen, each faculty has its own prerequisites that determine its own approach. Nevertheless, some cross-faculty success factors and obstacles could be identified, but with the caveat that the study is based on only four faculties and that only few people could be interviewed about the steps and aspects of the implementation. Nevertheless, a whole range of starting points for possible support programs emerged, some of which are relevant at different points in the process. As part of the further support of the faculties in their implementation process

## Funding

The study is part of the project “Communicative Competencies of Physicians – Pilot Implementation, Accompanying Evaluation, and Development of Implementation Strategies for a Longitudinal Model Curriculum Communication in Medicine”, which was funded by the German Federal Ministry of Health (funding code: ZMV I1 2516FSB200).

## Profiles

**Name of school: **German National Institute for state examinations in Medicine, Pharmacy and Psychotherapy (IMPP)

**Study program/occupation: **Medicine

**Number of students per year and/or per semester: **not applicable

**Has a longitudinal curriculum covering communication been implemented? **not applicable

**At which semester levels are communicative and social competencies taught? **not applicable

**Which teaching formats are used? **not applicable

**During which semesters are communicative and social competencies tested (formative, pass/fail, graded)? **State examinations

**Which assessment formats are used? **MC-questions, OSCE a.o.

**Who (e.g. hospital, institution) is in charge of development and implementation? **IMPP

## Current professional roles of the authors

Dr. phil. Barbara Hinding

- Graduate psychologist
- Research assistant at the German National Institute for state examinations in Medicine, Pharmacy and Psychotherapy (IMPP)
- Research areas: medical conversation and interprofessional communication in teaching and assessment, implementation of communication curricula in medical education and advanced training.

PD. Dr. med. Christian A. Brünahl

- Head of the department of further development of the examination system at the IMPP
- OSCE representative at the IMPP
- Senior physician at the University Medical Center Hamburg-Eppendorf.

Dr. med. Holger Buggenhagen, MME

- Senior physician in anaesthesiology
- Head of the central teaching platform Rudolf Frey Lernklinik at Mainz University Hospital
- The central teaching platform enables the implementation of curricular courses with a focus on the acquisition of skills in affective and psychomotor content areas as well as the realization of a medical didactics program, a program to improve the quality of teaching and the development of a new curriculum in Mainz.

Nadine Gronewold

- Psychologist (M.Sc.)
- Research assistant at Heidelberg University Hospital in the Department of General Internal Medicine and Psychosomatics
- Research areas: psychocardiology, e-health and development of interprofessional and communication-oriented medical education.

Dr. rer. physiol. Anke Hollinderbäumer, MME

- Graduate psychologist
- Research assistant at the Central Learning Platform Rudolf Frey Lernklink
- Responsible for the communication curriculum and OSCE examinations.

Dr. med. Kirsten Reschke, MME

- Senior physician at the Clinic for Nephrology and Hypertension, Diabetology and Endocrinology at the Medical Faculty of the University of Magdeburg
- Head of the Medical Didactics Working Group
- Teaching coordinator for internal medicine.

PD Dr. med. Jobst-Hendrik Schultz, MME (Bern)

- Senior physician, Heidelberg University Hospital, Clinic of General Internal Medicine and Psychosomatics
- Head of HeiCuMed (Heidelberger Curriculum Medicinale), Block Internal Medicine
- Head of MediKIT (Kommunikations- und Interaktionstraining in der Medizin) at the Medical Faculty Heidelberg.

Prof. Dr. med. Jana Jünger, MME (Bern)

- Director of the German National Institute for state examinations in Medicine, Pharmacy and Psychotherapy (IMPP)
- Developer of the post-graduation study program Master of Medical Education (MME), Germany
- Member of the MME-study program management and lecturer for the modules Assessment, Education Research and Evaluation
- Management of various programs on the implementation of communication curricula in medical training and the development of new examination formats for assessing communicative skills.

## Competing interests

The authors declare that they have no competing interests. 

## Figures and Tables

**Table 1:**
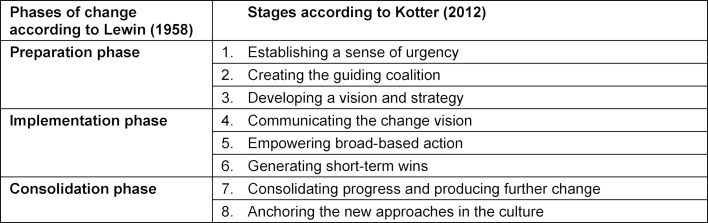
The eight steps of change management

**Table 2:**
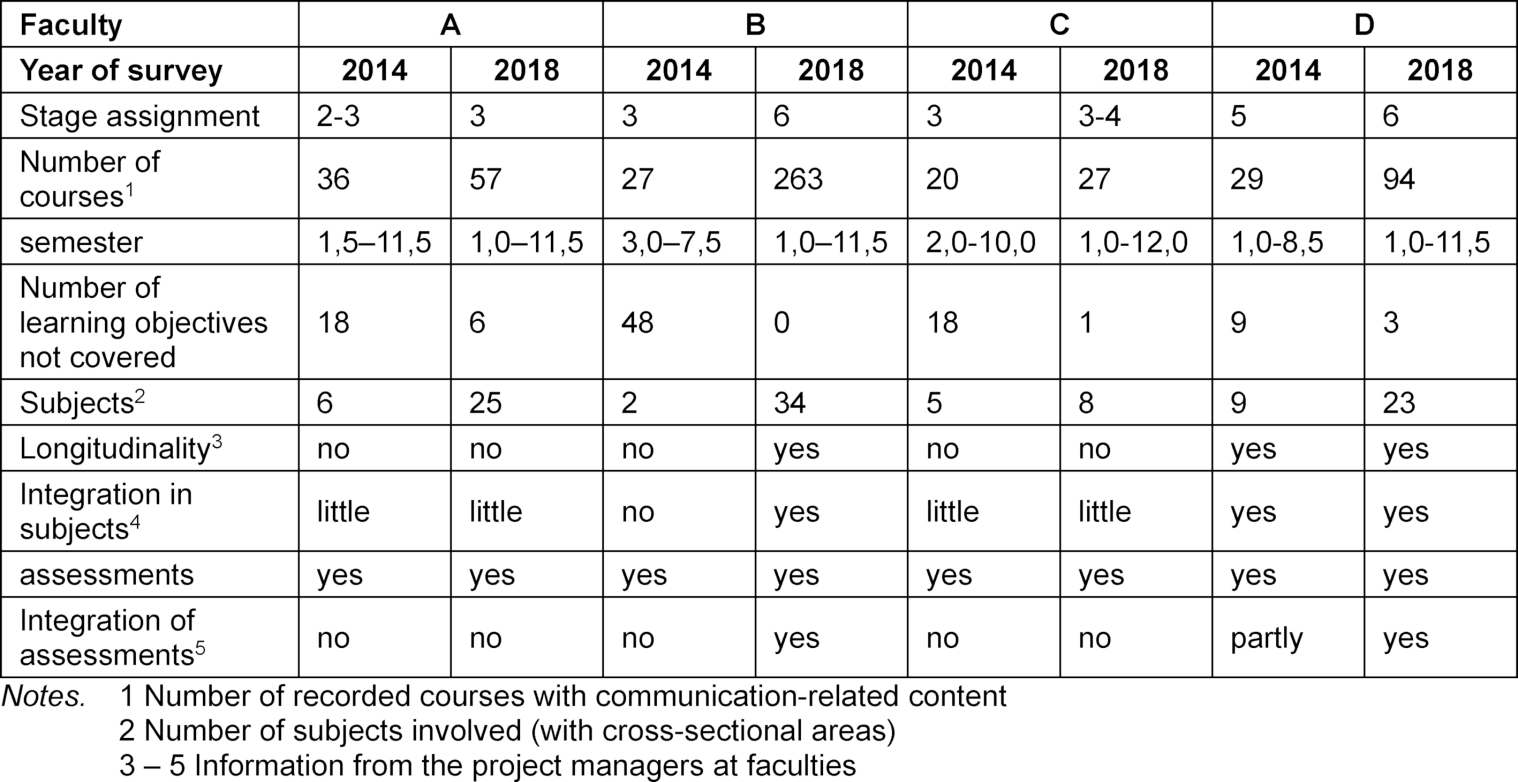
Before and after comparison of communication curricula

**Table 3:**
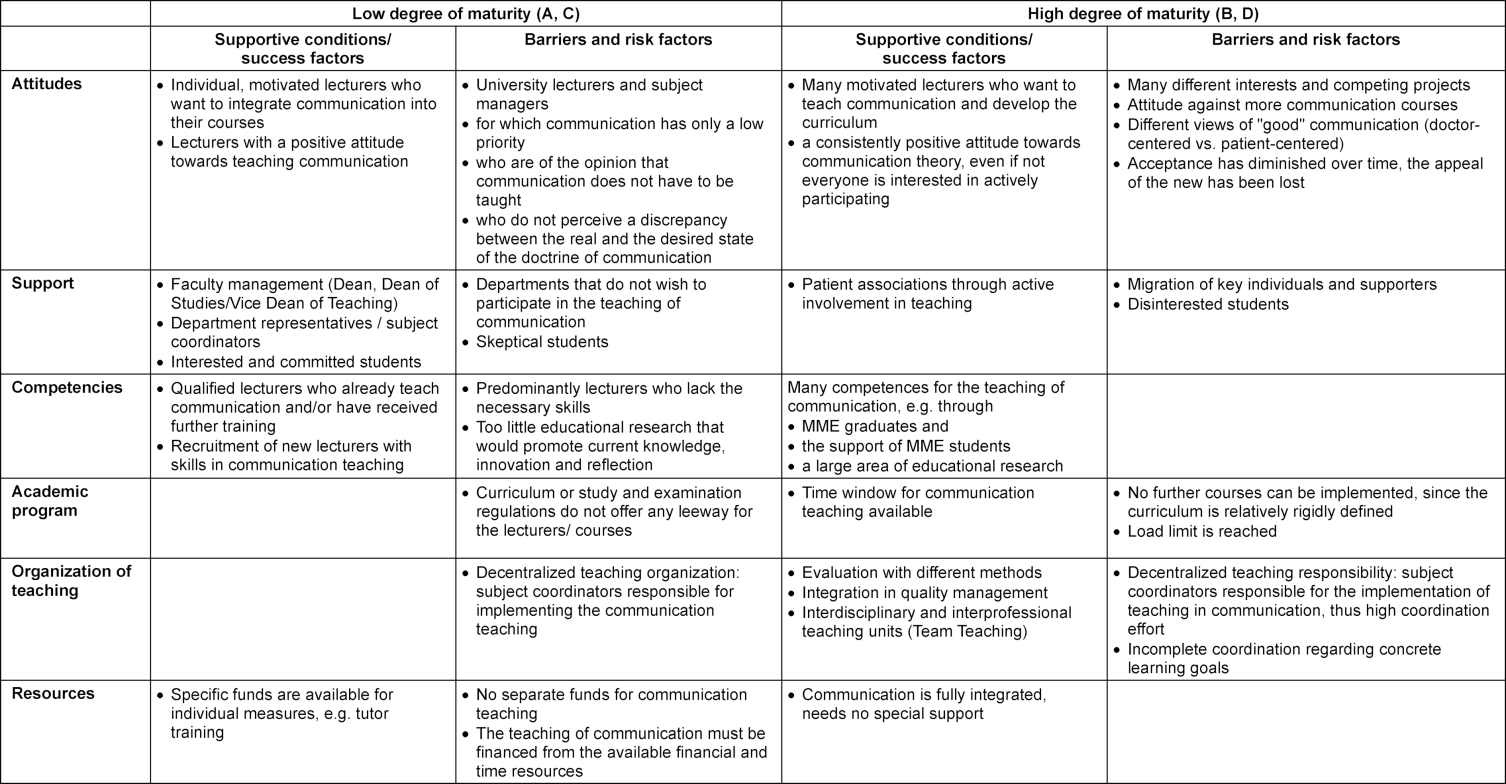
Promoting and inhibiting conditions in faculties with low and high maturity of the communication curriculum

**Table 4:**
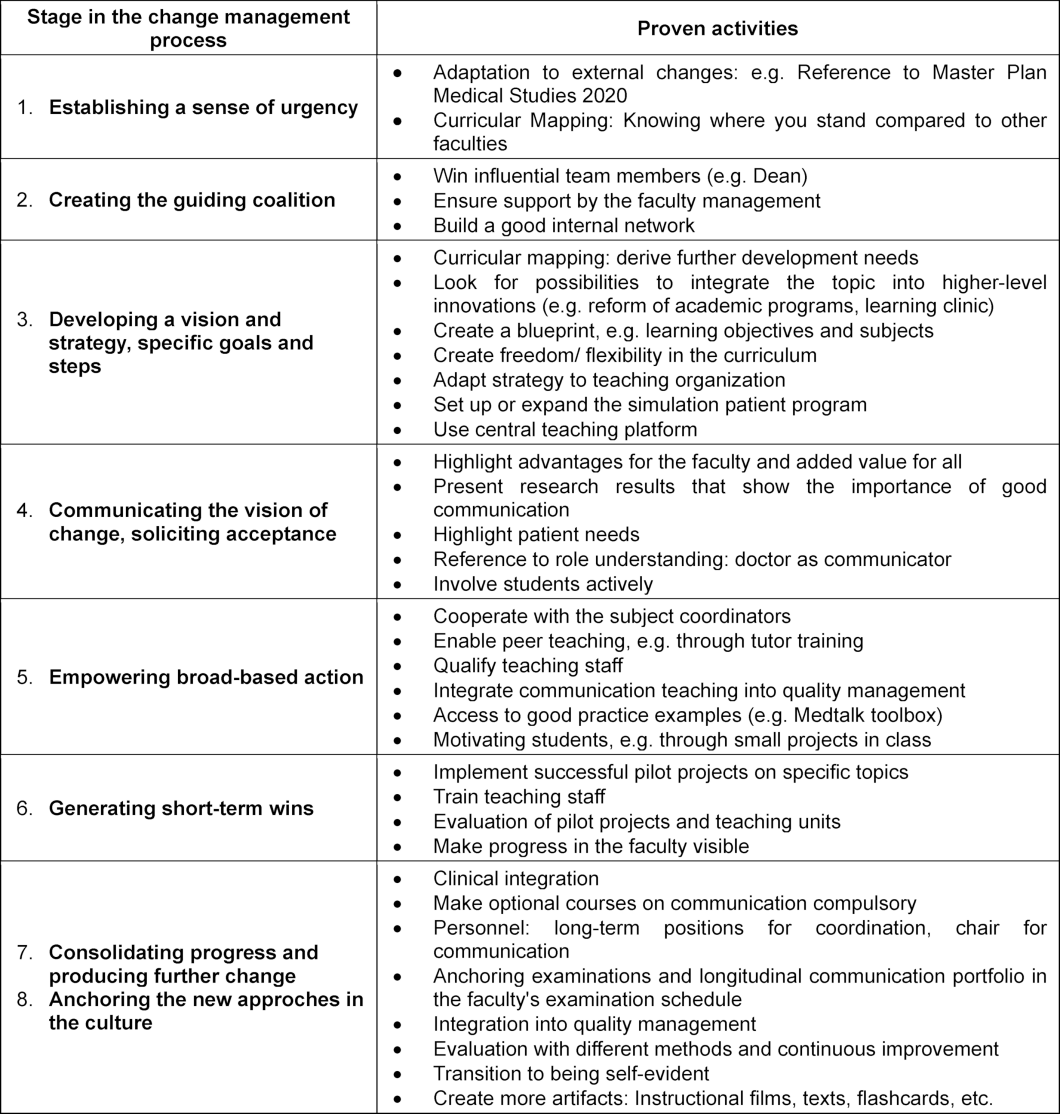
Proven activities and measures, assigned to the stages of change management

## References

[1] Stewart MA (1995). Effective physician-patient communication and health outcomes: a review. CMAJ.

[2] Thorne SE, Bultz BD, Baile WF, SCRN Communication Team (2005). Is there a cost to poor communication in cancer care?: a critical review of the literature. Psychooncology.

[3] Baile WF, Aaron J (2005). Patient-physician communication in oncology: past, present, and future. Curr Opin Oncol.

[4] Venetis MK, Robinson JD, Laplant Turkiewicz K, Allen M (2009). An evidence base for patient-centered cancer care: A meta-analysis of studies of observed communication between cancer specialists and their patients. Patient Educ Couns.

[5] Street RL, Makoul G, Arora NK, Epstein RM (2009). How does communication heal? Pathways linking clinician-patient communication to health outcomes. Patient Educ Couns.

[6] Kelley JM, Kraft-Todd G, Schapira L, Kossowsky J, Riess H (2014). The influence of the patient-clinician relationship on healthcare outcomes: a systematic review and meta-analysis of randomized controlled trials. PloS One.

[7] Jünger J, Köllner V (2003). Integration eines Kommunikationstrainings in die klinische Lehre. Beispiele aus den Reformstudiengängen derUniversitätenHeidelberg und Dresden. Psychother Psychosom Med Psych.

[8] Silverman DJ, Kurtz SM, Draper J (2013). Skills for Communicating with Patients.

[9] Mitzkat A, Schulz C, Kasenda B, Langer T, Schnell MW (2006). "ARZT IM GANZEN SPEKTRUM". Die INTEGRIERTEN CURRICULA der Medizinerausbildung an der Universität Witten/Herdecke - Rückblick auf sechs Jahre Lehre im Hinblick auf Praxisorientierung und theoretische Vorgaben. GMS Z Med Ausbild.

[10] Mortsiefer A, Rotthoff T, Schmelzer R, Immecke J, Ortmanns B, in der Schmitten J, Altiner A, Karger A (2012). Implementation of the interdisciplinary curriculum Teaching and Assessing Communicative Competence in the fourth academic year of medical studies (CoMeD). GMS Z Med Ausbild.

[11] Arends P, Breckwoldt J, Brunk I, Dettmer S, Kienle R, Hitzblech T, Hölzer H, Maaz A, Mossakowski A, Mühlinghaus I, Röhr C, Vogt K, Wendt O, Peters H (2012). Integration des Längsschnittcurriculums ,Kommunikation, Interaktion, Teamarbeit' (KIT) im Modellstudiengang Humanmedizin an der Charité Universitätsmedizin Berlin. Jahrestagung der Gesellschaft für Medizinische Ausbildung (GMA). Aachen, 27.-29.09.2012.

[12] Kiessling C, Langewitz W (2013). Das Longitudinale Curriculum "Soziale und kommunikative Komptenzen" im Bologna-reformierten Medizinstudium in Basel. GMS Z Med Ausbild.

[13] Görlitz A, Blum K, Feckl J, Pander T, Suda M, Fischer M, Kiessling C (2013). Implementierung eines longitudinalen lernzielbasierten Curriculums zur kommunikativen Kompetenz im Medizinischen Curriculum München (MeCuM).

[14] Sator M, Jünger J (2015). Von der Insellösung zum Longitudinalen Kommunikationscurriculum - Entwicklung und Implementierung am Beispiel der Medizinischen Fakultät Heidelberg. From Stand-Alone Solution to Longitudinal Communication Curriculum - Development and Implementation at the Faculty of Medicine in Heidelberg. Psychother Psych Med.

[15] Jünger J, Mutschler A, Kröll K, Weiss C, Fellmer-Drüg E, Köllner V, Ringel N (2015). Ärztliche Gesprächsführung in der medizinischen Aus- und Weiterbildung. Das Nationale longitudinale Mustercurriculum Kommunikation. Med Welt.

[16] Jünger J, Weiss C, Fellmer-Drüg E, Semrau J (2016). Verbesserung der kommunikativen Kompetenzen im Arztberuf am Beispiel der Onkologie. Ein Projekt des Nationalen Krebsplans. FORUM.

[17] Jünger J, Köllner V, von Lengerke T, Neuderth S, Schultz JH, Fischbeck S, Karger A, Kruse J, Weidner K, Henningsen P, Schiessl C, Ringel N, Fellmer-Drüg E (2016). Kompetenzbasierter Lernzielkatalog "Ärztliche Gesprächsführung". Z Psychosom Med Psychother.

[18] Universitätsklinikum Heidelberg (2016). Entwicklung des Nationalen longitudinalen Mustercurriculums Kommunikation in der Medizin.

[19] Reinmann G (2005). Innovation ohne Forschung? Ein Plädoyer für den Design-Based-Research-Ansatz in der Lehr-Lernforschung. Unterrichtswissenschaft.

[20] Barab S, Squire, K (2004). Design-Based-Research: Putting a Stake in the Ground. J Learn Sci.

[21] Hinding B, Deis N, Gornostayeva M, Götz C, Jünger J (2019). Patient handover - the por relation of medical training?. GMS J Med Educ.

[22] Silverman J (2009). Teaching clinical communication: a mainstream activity or just a minority sport?. Patient Educ Couns.

[23] Lewin K, Maccoby, EE, Newcomb TM, Hartley EL (1958). Group decision and social change. Readings in Social Psychology.

[24] Kotter JP (1996). Leading Change.

[25] Kotter JP (2012). Leading Change.

[26] Armenakis AA, Fredenberger WB (1997). Organizational change readiness practices of business turnaround change agents. Knowledge Process Manage.

[27] Armenakis A, Bedeian A (1999). Organizational change: A review of theory and research in the 1990s. J Manage.

[28] Holt D, Vardaman JM (2013). Toward a Comprehensive Understanding of Readiness for Change: The Case for an Expanded Conceptualization. J Change Manage.

[29] Kern DE, Thomas PA, Howard DM, Bass EB (1998). Curriculum Development for Medical Education: A Six-step Approach.

[30] Bohnsack R (2010). Rekonstruktive Sozialforschung.

[31] Bohnsack R, Przyborski A, Buber R, Holzmüller H (2007). Gruppendiskussionsverfahren und Focus Groups. Qualitative Marktforschung.

[32] Mayring P (2015). Qualitative Inhaltsanalyse, Grundlagen und Techniken.

[33] Fellmer-Drug E, Drude N, Sator M, Schultz JH, Irniger E, Chur D, Neumann B, Resch F, Jünger J (2014). Introducing a Curricular Program Culminating in a Certificate for Training Peer Tutors in Medical Education. GMS Z Med Ausbild.

[34] Ringel N, Bürmann BM, Fellmer-Drueg E, Roos M, Herzog W, Nikendei C, Wischmann T, Weiss C, Eicher C, Engeser P, Schultz JH, Jünger J (2015). Integrated peer teaching of communication and clinical skills how to train student tutors?. Psychother Psychosom Med Psychol.

[35] Mutschler A, Kröll K, Ringel N, Weiß C, Gornostayeva G, Fellmer-Drüg E, Jünger J (2016). Longkomm-Toolbox - Exchange of best practice examples on the subject of communication skills. https://www.researchgate.net/publication/323512666_Longkomm-Toolbox-_Exchange_of_best_practice_examples_on_the_subject_of_communication_skills.

[36] Harden R (2001). AMEE Guide No. 21: Curriculum mapping: a tool for transparent and authentic teaching and learning. Med Teach.

[37] Jacobs HH, Johnson A (2009). The curriculum mapping planner: Templates, tools, and resources for effective professional development.

[38] McLachlan JC (2006). The relationship between assessment and learning. MedEduc Online.

[39] Schuwirth L, van der Vleuten C (2004). Merging views on assessment. Med Educ.

